# Symmetric tilt grain boundaries in uranium nitride and uranium carbide

**DOI:** 10.1038/s41598-026-67229-y

**Published:** 2026-08-31

**Authors:** Cláudio M. Lousada

**Affiliations:** Department of Materials Science and Engineering, KTH Royal Institute of Technology, Stockholm, SE-100 44 Sweden

**Keywords:** Energy science and technology, Materials science, Physics

## Abstract

The development of UN as a nuclear fuel requires good understanding of its microstructure. Here is presented a study of a set of 14 symmetric tilt grain boundaries (GBs) in UN and UC using density functional theory (DFT). From this set, both for UN and UC, the Σ03 (111)[$\:\bar{1}$10] is the most stable GB. Correlations between properties of the GBs such as tilt angle, excess volume and energies were obtained. For both materials the GB energies have a global minimum at the tilt angle of 70°. The plot of the tilt angles vs. GB energies converges naturally to the previously published stacking fault energy for UN at 0°. Using this method, a stacking fault energy for UC was obtained. The correlation between GB excess volume and GB energy is strong for UN, it is single-valued and of the simple parabolic type and shows that the excess volume is a good predictor of the GB energy for this material. For UC, the corresponding correlation is moderately predictive. Similarly to what is known for MgO with rock salt structure, this set of GBs is expected to be a good description of coincidence site lattice GBs in UN and UC that occur in microstructures close to equilibrium.

**Supplementary Information:**

The online version contains supplementary material available at https://doi.org/10.1038/s41598-026-67229-y.

## Introduction

Uranium nitride is being considered as a candidate fuel for future nuclear reactors. The currently envisioned departure from UO_2_ is because there is a search for both accident tolerant fuels and for a material with higher fissile efficiency. Within ceramic fuels, materials that have one-to-one stoichiometry between the anion and the actinide cation have a higher fissile efficiency than that of UO_2_. At up to 5% ^235^U enrichment, the UN fissile efficiency is up to 40%^1,2^. Other properties of UN are also advantageous when compared to O based fuels. UN is less corrosive for the envisioned cladding materials^3^, has a higher melting *T*, and increased accident tolerance^4,5^. The metallic character of UN imparts a thermal conductivity to this material, ≈ 20 W/m·K at 1273 K which is also higher than that of UO_2_, ≈ 7 W/m·K^6,7^. Additionally, the occurrence of secondary phases and the placement of the eutectic points^8–13^, longer fuel cycle^1,14^, and excellent compatibility with the PUREX reprocessing method^14,15^ are advantageous when compared to UO_2_.

The development of UN as a fuel requires better knowledge of its properties. This includes the microstructure, as this parameter is essential for the performance of the material from different perspectives^16^. Diffusion and segregation of fission products, mechanical stability of the fuel, neutron absorptivities^17^, corrosion^18,19^, among other properties, depend on the microstructure of the material which suffers changes with burnup and at the different stages of the fuel cycle^20^. Even if other extended defects such as dislocations can have some relevance, the distribution and the types of grain boundaries (GBs) largely determine the microstructure of the material. In materials with fcc structure, the GBs can be of random high angle type or of coincidence site lattice (CSL) type. CSL GBs of the symmetric tilt type are usually used as models of GBs in fcc materials as these GB models provide a good description of the GBs observed in the materials^21^. For materials in or close to equilibrium, the GB distribution depends to good extents on the GB energy (γ_GB_). Consequently the frequency of occurrence of GBs correlates inversely with their γ_GB_^22^. For MgO which shares the rock salt structure with UN and UC, this has been verified^23^. For MgO, the γ_GB_ function has a broad minimum near the Σ03 misorientation^24^. In the same work the authors suggest that misorientations around the [100] axis and equivalents, create GBs with relative energies lower than those with misorientations around the [110] or [111] axes. A more recent study found that a set of GBs of the families [100] and [$\:\bar{1}$10] can be used as a good descriptor of GB properties in MgO^25^. An appropriate set of CSL GBs was able to grasp the diversity of coordination numbers, volume expansions, and local deviations from the symmetry of the bulk that exists in the large variety of GBs in the real material^25^. We have previously reported similar finds for fcc Cu and Al GBs^26–28^. Analyses of the sets of GBs using descriptors of symmetry were also reported in those works. The mathematical tessellation methods of the Voronoi or Laguerre-Voronoi types are essential to perform these analyses^26–28^. From these tessellation methods it is possible to derive symmetry quantifying parameters that can be used to describe the atomic structure at GBs with large detail. By comparing properties of the GB atoms with the corresponding quantities of the bulk atoms, we can obtain descriptive functions of the stability and chemical reactivity of the GBs based on structural parameters. We previously found that for fcc Cu and Al, quantities such as the average coordination number, or the overall excess volume used individually are functions that are not complete enough to be robust predictors of γ_GB_. This is because these functions are complex and are not single valued. Two parameters derived from the tessellation theory could however predict local properties of GBs of Cu and Al with good accuracy. These are: a parameter that quantifies the symmetry of the local atomic site; and the excess volume expressed as an average difference in volume of the GB atomic sites with respect to the volume of the bulk site (δ*V*)^26–28^. For UN and UC there are currently no studies of GBs where their energies and atomic structures are analysed with the level of detail that quantum mechanical modelling can provide.

Applying similar analyses for the case of a set of 14 GBs of the symmetric tilt type here reported for UN and UC, we observe that δ*V* can be used as a strong predictor of relative γ_GB_ for GBs in UN. For UC the correlation is moderate but still predictive. Similarly to MgO, the Σ03 GBs are the most stable GBs in UN and UC and thus are those that are expected to occur most often in materials whose microstructures are close to or in equilibrium.

## Computational methods

### Static DFT

DFT calculations of the GB structures and energies were done with the Vienna ab initio simulation package (VASP 6.4.2)^29^ using the Perdew-Burke-Ernzerhof^30,31^ (PBE) exchange-correlation functional with pseudopotentials consistent with the projector augmented wave^32,33^ (PAW) type. Gaussian smearing with a width of 0.05 eV was employed. This width is considerably smaller than what is encountered in the literature for UN. Gaussian smearing avoids the non-monotonical occupation issue encountered with Methfessel-Paxton smearing which can cause the chemical potential to not be uniquely defined^34^.

The PBE functional is able to describe the structure of high-angle low-index symmetric tilt GBs of metals and of rock salt structure materials with good accuracy^25,35–39^. Given that UN has metallic character, this functional was employed for the study of UN and UC GBs after evaluation of its performance both against literature data that used this functional and the own tests here reported^40,41^. We performed tests of PBE and of the Hartree-Fock DFT functionals PBE0 and HSE06, and of PBE with added Hubbard correction for strongly correlated systems in the framework of DFT + U^42^, as implemented in the VASP code^43^. The DFT + U methodology here employed used parameters previously benchmarked^44–46^ and that consist of the rotationally invariant approach by Dudarev^47^ with effective on-site Coulomb interactions (U_eff_ = 1.9 eV) and effective on-site exchange interactions (J = 0.1 eV) for a total U = 2.0 eV. For the tests of the functionals, all parameters besides the functional specific parameters were kept equal.

For describing the magnetic states in the bulk crystals, spin-orbit coupling within the non-collinear framework of VASP was used in all cases^48^. For all cases, f-orbitals were considered in the one-center PAW charge densities and added to the density mixer. A plane wave cutoff of 520 eV was employed, together with k-point meshes of (4 × 4 × 4) and (10 × 10 × 10) in the Monkhorst-Pack sampling scheme^46^. Because of the convergence between these two meshes (in the order of 0.01 eV in the electronic energies), the (4 × 4 × 4) mesh was employed for data production. For the bulk case, the simultaneous optimization of the unit cell volume, shape and internal atomic coordinates was carried out to understand the effect of different magnetic states on the cell size and shape. The initial guess magnetic moment of the U-atoms used as input was retrieved from experimental data from the literature^49^. From there the supercell sizes and shapes were optimized with simultaneous optimization of the spin using the VASP non-collinear solver without any constraints.

Based on the results for the different functionals that will be discussed later in this text, the PBE functional was used for the study of the sets of GBs. For the GBs, k-point meshes of (3 × 5 × 7) in the Monkhorst-Pack sampling scheme were chosen based on our previous studies of GBs where the choice of the k-point meshes considered both computational performance and accuracy^26,28,36,50^. The static DFT energies—that will be referred to as energies from now onwards—are electronic energies at 0 K. These energies allow accurate comparisons between local atomic structures of sites with similar chemical environment, without the need to include the computationally prohibitive vibrational contributions^51–53^. The γ_GB_ are thus defined as

1 $${\gamma _{GB}}={\text{ }}({E_{GB}}-{E_{bulk}})/2A$$

where *E*_GB_ is the energy of the supercell that contains the GB, *E*_bulk_ is the total energy of a supercell of UN or UC single crystal with the same number of atoms as those present in the GB supercell, and *A* is the area of the GB plane in the supercell. The division by two is applied because each supercell used to model a GB contains two identical GBs due to periodicity. A lower γ_GB_ implies stronger bonding and consequently stronger cohesion between the two grains.

The stoichiometry of the materials has been maintained in every GB model here used. The geometry optimizations were done first for the atomic positions keeping the supercell sizes fixed after which the supercell sizes were optimized, followed by another optimization of the atomic positions until the Hellmann–Feynman force acting on each atom was less than 0.01 eV·Å^-1^ and the total energy converged below 1 × 10^− 5^ eV.

Tests of the optimization of the magnetic configuration of the Σ03 (111)[$\:\bar{1}$10] GB in UN were performed with DFT + U and the modelling parameters described in the methods section. The ferromagnetic (FM) and antiferromagnetic (AFM) configurations were compared. In both cases the functional found a ground state FM magnetic configuration with noncollinear spin orientations at the GB plane. The γ_GB_ for the FM configuration is 1052 mJ/m^2^ which is lower than the value obtained with PBE for a non-magnetic structure, γ_GB_ = 1436 mJ/m^2^. However, the result is inconclusive regarding the magnetic structure of the GB because the model used could not predict the correct magnetic configuration at the bulk section of the GB model. When using periodic supercells to model CLS GBs it is imperative that with increasing distance along the perpendicular to the GB plane, the properties of the GB model start to approach those of the bulk. Typically, at around 1/3 and 3/4 of the supercell length, the bulk atomic properties are verified. In the case of the magnetic properties, these were not verified due to the spin constraints that are imposed on these bulk atoms by the proximity to the GB atoms. This is not representative of the real system and thus we conclude that the models here used do not allow the study of magnetic properties of the GBs. Considerably larger models that are computationally prohibitive must be employed, or the magnetic structure should be optimized with spin constraints at the sections of the models that represent the bulk after a careful development of the methodology. The geometric and energetic properties (other than magnetic) of the GBs could be correctly modelled with the models here employed.

### Ab initio molecular dynamics simulations (AIMD)

Ab initio molecular dynamics simulations (AIMD) were performed on two of the GBs with the lowest γ_GB_ according to the static DFT results for UN and UC. These simulations were done to understand the role of vibrations due to finite temperature in the stability of the models and to compare their relative energies. Some defects can give rise to new vibrational modes with larger momenta than those that occur in the single crystal^54^. If this occurs, the resulting GB free energy could change and the GB models could become unstable especially at high *T*. The DFT optimized structures were simulated with AIMD both at 298 K and 1700 K using the canonical (*NVT*) ensemble in the code VASP with the PBE functional. The same models and electronic structure parameters as described in Sect. 2.1 were used. For the dynamics, the temperature was controlled with the Nosé-Hoover thermostat^55,56^, with a Nosé mass corresponding to a Nosé frequency of 280 cm^-1^, and a time-step of 8 fs at 298 K and of 3 fs at 1700 K which are sufficient to describe the vibrational motion in UN and UC with accuracy^57^. The systems were first well equilibrated in simulations of around 1.5 ps after which the statistics were collected for typically 4.5 to 6.5 ps of simulation time.

The AIMD free energy is equivalent to the Helmholtz free energy (*F*) of an ensemble and was obtained from

2 $$\:F=-{k}_{b}T\mathrm{l}\mathrm{n}Q$$

where *Q* is the partition function defined as a phase space integral of all spatial and momentum coordinates. For two systems that are similar enough, the free energy of a structure resulting of a transformation, ($\:\bar{{F}_{r}}$)—which is the difference between the free energies of a product or final configuration (*F*_*p*_) and that of a reactant or initial configuration (*F*_*r*_)—is given by

3 $$\:\left( {\bar{F}_{r} } \right) = \left\langle {F_{p} } \right\rangle - \left\langle {F_{r} } \right\rangle = k_{B} \ln \left( {\frac{{\left\langle {e^{{E/k_{B} T}} } \right\rangle _{p} }}{{\left\langle {e^{{E/k_{B} T}} } \right\rangle _{r} }}} \right)\:\:\:$$

in the case of similar systems, the ratio of the expectation values in Eq. 3 is close to unity and the errors of the ensemble averages cancel to a large extent leaving only a residual error. In this framework, the approximations for the determination of *F* used in VASP cancel largely for *F*_*p*_ and *F*_*r*_ and the AIMD free energies correspond to the VASP free energies obtained in the simulations. The free energies of the GBs (γ_GB_AIMD_) were determined with Eq. 1 but where instead of the electronic energies *E*, the average free energies ($\:\bar{F}$) were employed.

### Structural analyses

To understand the effects of the local geometry in the properties of the GBs, structural analyses were carried out following our previously published methods that use symmetry descriptors^26–28^. Spatial tessellation methods allow the analytic determination of space groups for non-trivial cases^58,59^. Our previous work with tessellation methods for structural analyses of this kind was done for GBs of metals^26–28^. Other authors have demonstrated the usefulness of tessellation methods for other types of studies^36,59^. Because of the size differences between the N or C-atom and the U-atom, Laguerre-Voronoi diagrams were obtained taking into account the differences in the atomic radii. In the Laguerre-Voronoi tessellation, the van der Waals radii of N, C and U were employed. The polyhedra generated by tessellation of the supercells were used to determine the volume expansion (*V*_x_) of GB atomic sites. The *V*_x_, of a GB site is here defined as the volume of the U-atom at the GB site relative to the volume of the U-atom at the bulk reference site. Following a method previously published^28,60^, the total excess volume at the GBs (δ*V*) with the units of Å were determined as.

 

4 $$\delta V{\text{ }} = \:\left(\frac{{V}_{tot}-{N{\Omega\:}}_{bulk}}{2A}\:\right)$$

 

where *V*_*tot*_ is the total volume of the GB, *N* is the number of atoms in the supercell, Ω_*bulk*_ is the volume per atom in the bulk crystal and *A* is the area of the GB plane in the supercell. *V*_*tot*_ was obtained from the average *V*_*x*_ at the GB atoms and this quantity is thus a GB excess volume expressed per GB atom, considering only the GB atoms that had a *V*_*x*_ ≠ 0 relative to the bulk, reference atom.

### GB models

The GB models used were constructed from known defects that occur in MgO that also has a rock-salt structure and where the anion is an early p-block element as in the cases of UN and UC. Because of the type anion in these materials the relative energies of their different extended defects are expected to follow similar trends^61,62^.With basis on the GB models for MgO, CSL symmetric tilt GB models of UN and UC were constructed using a methodology that has been thoroughly benchmarked and tested in previous studies^27,37,38^. The CSL symmetric tilt GBs are constructed by creating a supercell with to oppositely oriented GBs tilted with a defined tilt angle about a defined tilt axis creating an interface between grains along a plane that is a unique solution of the two parameters tilt angle and tilt plane for each crystal structure: fcc, bcc, hcp etc. In polycrystals, the GBs are not exactly of the CSL type, but the properties of their GBs are also verified in GBs with misorientations close to those of the CSL type^63^. Because of this, the CSL method is a good approximation to model symmetric tilt GBs that occur in real materials^63–65^. The models here employed were constructed taking into account parameters for the choice of supercell size previously devised for modelling these defects with DFT^26,63^. The models have only the minimum number of unique atoms that are needed to describe the GB in terms of symmetry. After construction, the supercell models were optimized according to the procedure described in Sect. 2.1 with optimizations of their volume and internal atomic coordinates. The fitting of the mathematical functions to obtain correlations between properties of the GBs was done with MATLAB R2024b^66^. The statistical errors of the goodness-of-fit for each function are provided in the Supporting Information (SI) section.

### Correlation between GB tilt angle and GB energy, and relation with the stacking fault energy (SFE)

The Read-Shockley model is a relationship between the GB energy (γ_GB_) and the angle (*θ*) between the adjacent grains^67^. The model has been derived assuming that GBs can be described by a dislocation model previously proposed by Burgers and Bragg^68,69^, and it is applicable to simple cubic lattices with isotropic elasticity and small misorientations where the grains are rotated about an axis in a two-dimensional model of the GB. Mathematically, the Read-Shockley model is expressed as

5 $${\gamma _{GB}}\,=\,{\gamma _0}\theta (A-\ln \theta )$$

where *A* is a trigonometric function of the angles of the planes that define the dislocation model of the GBs, and that are tilted about the reference axis. The *A* function has also a parameter that accounts for the energy due to atomic misfit as a function of the angle^67^. *A* has also a zero term

6 $${A_0}\,=\,1{\text{ }}+{\text{ }}\ln (a/2\pi {r_0})$$

In this expression *a* is the spacing between dislocations that constitute one unit length of the GB, and *r*_0_ is obtained from the expression for the variation of the elastic energy density for a single dislocation—which varies as (1/*r*^2^) from the center of the dislocation—and it is a logarithmic infinity if integrated to *r* = 0. In Eq. 5, γ_0_ depends only on the orientation of the GB and on the macroscopic elastic constants such as the shear modulus and Poisson’s ratio. The Read-Shockley model has experimental accuracy for GBs with *θ* ≤ 15°. Equation 5 can be rearranged to linear form as

7 $$\:\frac{{{\upgamma\:}}_{GB}}{{{\upgamma\:}}_{0}\theta\:}=A\:-\:ln\:\theta\:$$

The intercept at origin is 0 = *A* – ln 0 = *A*_*0*_. In this model, γ_0_ captures all long-range macroscopic elastic strains and *A*_0_ represents the short-range, non-elastic atomic distortion at the center of the dislocation core according to Eq. 6. Furthermore, in the original paper by Read and Shockley, the ratio γ_GB_/γ_0_ has a positive intercept at origin in the non-logarithmic plot of γ_GB_/γ_0_ *vs. θ*^67^. γ_0_ can thus be interpreted as representing the strength of the long-range stress field generated by the dislocations that constitute the GB. The ratio γ_GB_/γ_0_ is the GB energy expressed per unit GB plane area, normalized by these elastic and structural properties that depend on the lattice constant and composition of the material. The plot of γ_GB_/γ_0_ *vs. θ* in the Read-Shockley model is not enforced to be zero when *θ* = 0^67^. When *θ* = 0, the ratio γ_GB_/γ_0_ does not include the tilt angle of the dislocations that constitute a GB, but it is still normalized by the elastic energy density for a single dislocation plane due to the division by γ_0_ that considers the shear modulus and Poisson’s ratio of the corresponding unit area. The Read-Shockley model has been more recently expanded to high angle GBs^70^ for which its predictive power is more limited than for GBs with tilt angles *θ *≤ 15°^70^. This is because as the tilt angle increases so increases the number of solutions to the GB structure in terms of expressing this defect as sets of dislocations.

If the relation between γ_GB_ and tilt angle *θ* is expressed directly, i.e. without normalization by the parameter γ_0_ that includes the macroscopic elastic constants; the quantity being correlated with *θ*, γ_GB_ only, has not been normalized by the contribution of these elastic properties. This implies that those contributions are now implicitly included in d*γ*_*GB*_/d*θ*. Stacking faults (SF) occur due to the dissociation of lattice dislocations into partial dislocations. A process that reduces the elastic energy via a mechanism that follows Frank’s rule. The stacking fault energy (SFE) quantifies the energetic cost of shearing one atomic plane with respect to another atomic plane. This process is thus directly related to the response of a crystal lattice to deformation. More specifically, the SF occur when a perfect dislocation splits into partial dislocations that repel each other elastically creating a gap between them. The creation of a SF increases the energy of the crystal. The SFE correlates with elastic properties such as the bulk modulus and the equilibrium volume of the SF^71^, which in turn depend on the chemical composition of the material and on its lattice constant^72^.

In a direct correlation between γ_GB_ and *θ*, i.e. not-normalized by *γ*_0_ as in the case of the Read-Shockley law where *θ* is correlated with γ_GB_/γ_0_; elastic and structural properties of the crystal that are otherwise normalized by γ_0_ such as the shear modulus, Poisson’s ratio, and the misorientation between crystals which is related to the volume of the GB, are now included in the correlation. The classical Read-Shockley model treats the crystal as a linearly elastic continuum: the atoms that are displaced from their single crystal lattice positions to form a GB have interaction forces that follow a linear stress-strain relationship. This is a generalized equivalent of Hooke’s law. Hence, functions of quadratic nature are expected to describe the correlation between γ_GB_ and tilt angle. Thus, if the function γ_GB_(*θ*) is quadratic, when *θ =* 0, the value of γ_GB_ is due to the elastic energy of the smallest area of dislocation plane possible in the crystal, which is the quantity equivalent to the energetic cost of shearing one atomic plane, which in turn is a quantity closely related to the SFE. In crystals with fcc structure, symmetric tilt GBs have a core that structurally resembles an intrinsic SF both in terms of atomic coordination and very often in terms of excess volume^73^. It can thus be argued that in such conditions, γ_GB_ (*θ = 0*) ≈ SFE.

**Fig. 1:**
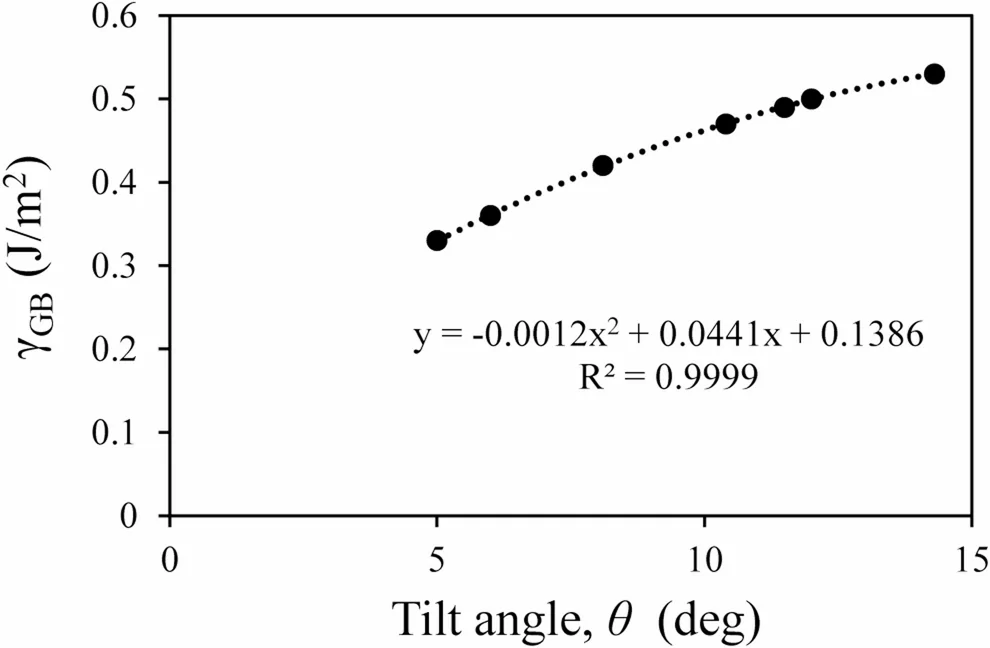
Correlation between GB energy (γ_*GB*_) and corresponding GB tilt angle (*θ*) for GBs of fcc Al for *θ* < 15° after data from reference^74^. The intercept at origin is a quantity close to the stacking fault energy according to the model here proposed.

The validity of this principle was here tested using a literature dataset for GBs in fcc aluminum, considering the data for γ_GB_ and the corresponding tilt angles^74^. The dataset was used to plot the data shown in Fig. 1, including only the GBs with *θ* < 15° to obey the criteria of applicability of the Read-Shockley model. The correlation between γ_GB_ and tilt angle *θ* has been verified to be a function of the type

8 $${\gamma _{GB}}\,=\,a{(\theta )^2}+b(\theta ){\text{ }}+{\gamma _0}$$

where *a* and *b* are fitting parameters and γ_0_ is the γ_GB_ value at (*θ = 0*). Fitting this function to the data of reference^74^ gives γ_GB_ = −0.0012(*θ*)^2^ + 0.0441(*θ*) + 0.1386, with an R^2^ = 0.9999.

The intercept at origin is ≈ 0.139 J/m^2^ which is in excellent agreement with the SFE of fcc Al 0.124 J/m^2^ obtained from modelling and 0.166 J/m^2^obtained from experiments^75^^,76^. A similar correlation was devised for UN including all GBs of the set here studied with angles smaller than the inflection point at 70.53°. The SFE obtained with this method is in excellent agreement with literature data as it will be discussed below and shows that this model allows the determination of the SFE for UC from the quadratic function *γ*(*θ*) when *θ* = 0.

## Results and discussion

### Bulk UN and UC

The equilibrium sizes and shapes of the supercells of single crystal UN and UC were determined for different ferromagnetic (FM) and antiferromagnetic (AFM) configurations, using as initial guess the experimentally determined magnetic moment for the U-atoms (M_U_). The directions [001] and [111] for the initial M_U_ were investigated as input parameters. Because in these materials the magnetic moments of the N and C-atoms are small and their determination with DFT is challenging, they are not here considered in the discussion of magnetism. The resulting data are shown in Tables 1 and 2 for UN and UC respectively.

**Table 1:** Tests of different DFT functionals for bulk UN single crystal: equilibrium lattice parameter (Å), equilibrium lattice angles (degrees), total electronic energy (eV) of the unit cell, total magnetic moment magnitude *M*_tot_ (Bohr magnetons (μ_B_) per unit cell), average magnitude of the local magnetic moments at the U atoms M_U_ (μ_B_ per atom).

Quantity	UN								
	Exp.	PBE (FM)	PBE (AFM)	PBE0 (FM)	PBE0 (AFM)	HSE06 (FM)	HSE06 (AFM)	DFT + U (FM)	DFT + U (AFM)
Lattice parameters: a, b, c (Å)	a, b, c = 4.890^77^	a, b, c = 4.873	4.876 4.867 4.876	a, b, c = 4.872	4.869 4.880 4.869	a, b, c = 4.873	4.869 4.881 4.869	a, b, c = 4.873	4.871 4.877 4.871
Lattice parameters: α, β, γ (deg)	α, β, γ = 90.000^77^	α, β, γ = 89.993	90.023 90.056 90.023	α, β, γ = 89.8485	90.051 90.198 90.051	89.841 89.841 89.841	90.055 90.192 90.055	89.885 89.885 89.885	90.059 90.286 90.059
Energy (eV)	–	−99.712	−99.615	−126.473	−126.378	−119.146	−119.066	−93.536	−93.638
*M*_tot_ (μ_B_)	–	4.737	0.008	5.108	0.178	5.231	0.004	4.810	0.012
*M*_U_ (μ_B_)	0.750^49^	0.729	0.959	1.351	1.409	1.3821	1.408	1.289	1.418

The AFM configuration was correctly predicted as energetically more stable than the FM by the DFT + U method, with a small energy difference between the two configurations. The *M*_U_determined with PBE is in excellent agreement with experimental results while the corresponding value obtained with DFT + U deviates considerably, by ≈ 50%^49^. These findings are in agreement with previous reports^46^. All functionals correctly predict a *M*_tot_ larger for the FM configuration than for the AFM. However, the lowest energy magnetic configurations were found to be FM or ferrimagnetic for UN with all functionals except the DFT + U. This considering that the *M*_tot_ obtained with PBE and DFT + U are ≈ 0 and thus correspond to AFM. The magnitudes of *M*_tot_ for each magnetic configuration are similar for all functionals. The PBE functional fails to predict the AFM configuration as the ground state, but the difference with the energy of the FM is very small: *E*_AFM_ - *E*_FM_ = 0.097 eV per unit cell which means 0.0243 eV/stoichiometric unit of UN that corresponds to 2.328 kJ/mol of UN. In general the fact that these two structures are close in energy agrees with the low Néel temperature of UN = 59 K—for the transition to the paramagnetic state—which in turn is consistent with the small *M*_U_ = 0.750 μ_B_^49^. The low value of the Néel temperature implies that different magnetic structures such as the FM and the AFM are close to degenerate as discussed in the original experimental work^49^. This is typical of itinerant ferromagnets that according to the Hills rule for uranium ceramics have U-U distances around or below the Hill limit: 3.4 Å^78^. Additionally, the difference in the energies between the AFM and the FM configurations is in excellent agreement with a recently published work^46^. The lattice parameters predicted by the different functionals deviate very little from the experimental value. For the cases where the functionals predicted non-orthorhombic unit cells, the deviations from the orthorhombic structure are extremely small. These findings show that optimizing the unit cell parameters without symmetry constraints while searching for the ground state magnetic structure can produce data very close to experiments with the PBE functional. This procedure together with the inclusion of spin-orbit coupling in a noncollinear spin framework without constraints is essential for the prediction of the correct magnetic solution for UN from electronic energies obtained from DFT + U.

Because the differences in energy between AFM and FM configurations obtained with PBE are so small and because the functional predicted correctly the magnetic moment in the U-atoms in the AFM conformation in better agreement with experimental data than the DFT + U method, we employed the PBE functional to study the GBs in UN.

**Table 2:** Tests of different DFT functionals for bulk UC single crystal: equilibrium lattice parameter (Å), equilibrium lattice angles (degrees), total electronic energy (eV) of the unit cell, total magnetic moment magnitude *M*_tot_ (Bohr magnetons (μ_B_) per unit cell), average magnitude of the local magnetic moments at the U atoms M_U_ (μ_B_ per atom).

Quantity	UC								
	Exp.	PBE (FM)	PBE (AFM)	PBE0 (FM)	PBE0 (AFM)	HSE06 (FM)	HSE06 (AFM)	DFT + U (FM)	DFT + U (AFM)
Lattice parameters: a, b, c (Å)	a, b, c = 4.957^79^	a, b, c = 4.959	a, b, c = 4.959	4.966 4.945 4.966	4.963 4.950 4.963	4.964 4.944 4.968	4.967 4.943 4.966	a, b, c = 4.959	a, b, c = 4.956
Lattice parameters: α, β, γ (deg)	α, β, γ = 90^79^	α, β, γ = 90.000	90.000 89.988 90.000	89.889 89.933 89.829	89.840 89.941 89.842	90.011 90.005 90.084	89.799 89.932 89.854	α, β, γ = 90.000	α, β, γ = 89.997
Energy (eV)	–	−94.007	−94.010	−118.211	−118.214	−110.610	−110.612	−87.306	−87.591
*M*_tot_ (μ_B_)	0.000^79^	0.000	0.000	0.130	0.007	0.967	0.028	0.003	0.000
*M*_U_ (μ_B_)	–	0.030	0.034	1.830	1.406	1.39	1.414	0.048	0.835

For UC, the AFM state was predicted by PBE and DFT + U to be slightly lower in energy than the FM state. Starting with the FM configuration as input led still to a slightly disordered AFM configuration in agreement with experimental data^79^. The PBE0 functional predicted the existence of a ferrimagnetic configuration with small *M*_tot_. The HSE06 functional failed to predict a correct magnetic solution consistent with the symmetry of the crystal. The lattice parameters predicted by PBE and DFT + U are extremely close to the experimental values. The data also shows that the results from DFT + U and PBE for UC are consistent both with experimental data and with the Hills rule^79^. With basis on these results the PBE functional was used for the study of the GBs of UC.

### Symmetric tilt grain boundaries (GBs) in UN and UC

**Fig. 2:**
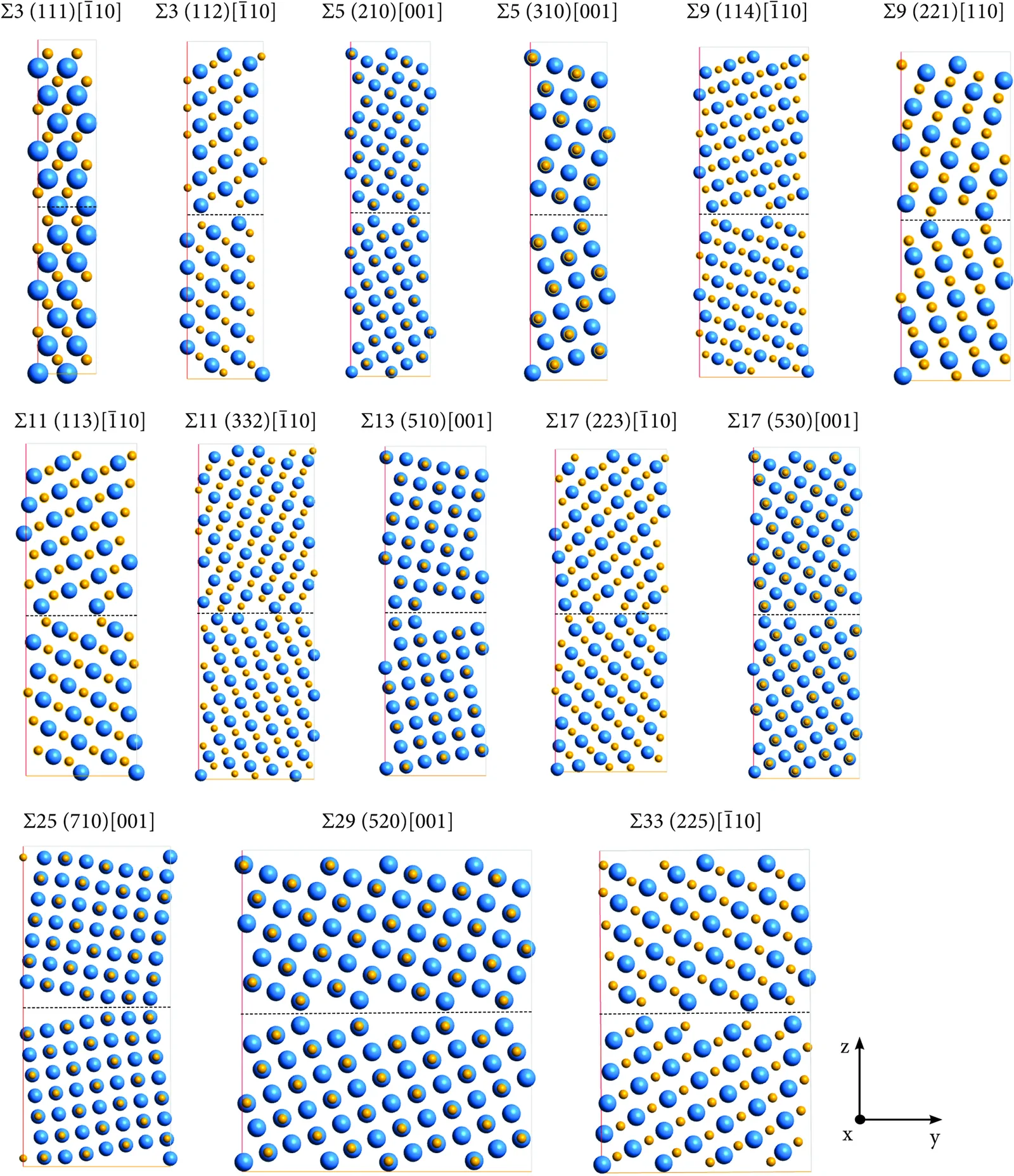
Set of symmetric tilt GBs of UN and UC studied in this work. Uranium (blue), N or C (yellow). The black dashed lines at the center of the supercells represent the GB planes.

The structures of the set of GBs were optimized with PBE and spin polarized calculations. The GB models of UN and UC are shown in Fig. 2 and their properties are summarized in Table 3.

**Table 3:** Properties of the models of the symmetric tilt GBs of UN and UC here studied with static DFT. GB notation: plane (hkl), rotation axis [uvw].

GB type	Tilt angle (degrees)	*n* atoms	UN		UC	
			γ_GB_ (mJ/m^2^)	Area (Å^2^)	γ_GB_ (mJ/m^2^)	Area (Å^2^)
Σ11 (113)[$\bar{1}$10]	129.52	88	2742	39.66	1869	40.55
Σ11 (332)[$\:\bar{1}$10]	50.48	176	2313	56.17	1171	56.64
Σ13 (510)[001]	22.62	156	2435	60.23	1740	62.02
Σ17 (223)[$\:\bar{1}$10]	93.37	136	2229	49.40	1595	49.97
Σ17 (530)[001]	61.93	204	2524	70.26	2310	71.36
Σ25 (710)[001]	16.26	200	2169	83.41	1538	86.21
Σ29 (520)[001]	43.6	232	2728	128.10	2313	129.81
Σ33 (225)[$\:\bar{1}$10]	121.52	132	2824	69.07	2307	70.13
Σ03 (111)[$\:\bar{1}$10]	70.53	48	1436	20.64	656	20.91
Σ03 (112)[$\:\bar{1}$10]	109.47	72	2480	29.51	2261	29.69
Σ05 (210)[001]	53.13	160	2549	53.45	2283	53.70
Σ05 (310)[001]	36.87	80	2691	37.55	2154	38.19
Σ09 (114)[$\:\bar{1}$10]	141.06	144	2772	50.84	2124	51.44
Σ09 (221)[$\:\bar{1}$10]	38.94	72	2337	35.80	1571	37.30

**Fig. 3:**
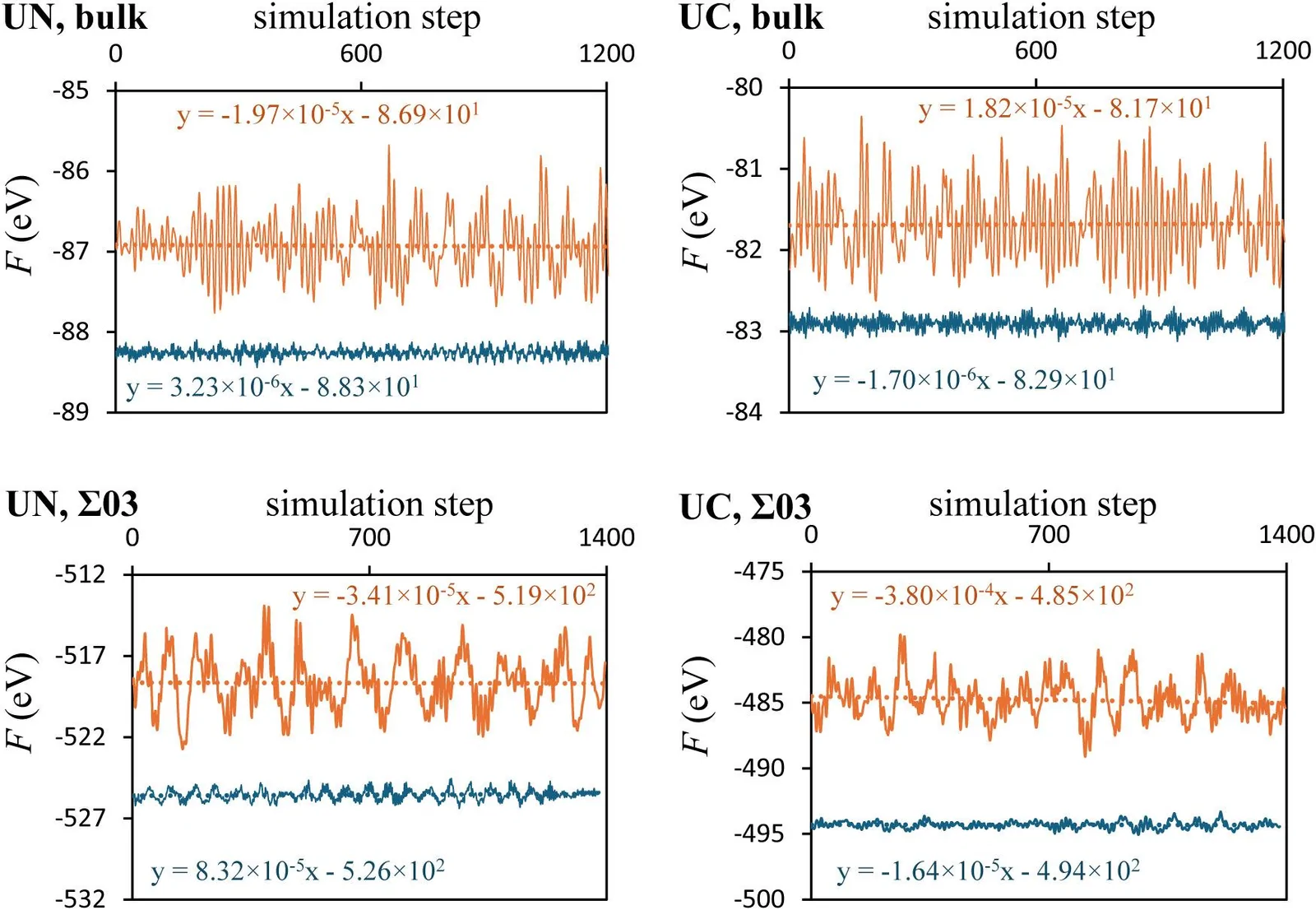
Free energy (*F* (eV)) vs. simulation step in AIMD simulations of the Σ03 (111)[$\:\bar{1}$10] GBs of UN and UC at 1700 K (orange) and 298 K (blue). Dotted lines are the least squares fit to the corresponding data. The uncertainties in *F* are given by the slopes. The time steps used are 8 fs for 298 K and 3 fs for 1700 K.

The Σ03 (111)[$\:\bar{1}$10] GB structures optimized with static DFT were studied with AIMD to verify their finite *T* stability. The structures are stable at the temperatures studied, *T* = 298 K and 1700 K. The *F* as a function of simulation step during the collection of statistics are shown in Fig. 3. *F* related properties are given in Table 4.

**Table 4:** Free energy (*F*) related properties obtained with AIMD for simulations of the Σ03 (111)[$\:\bar{1}$10] GBs of UN and UC. The uncertainties (*δE*_tot_) are given by the coefficients of the least square fits to the simulation data shown in Fig. 3. The statistical variances of *F* are also shown.

	UN		UC	
T	298 K	1700 K	298 K	1700 K
*F* (eV)	−525.550	−518.668	−494.303	−484.771
*δ F*_tot_ (eV/step)	± 8.320 × 10^− 5^	± 3.410 × 10^− 5^	± 1.640 × 10^− 5^	± 3.800 × 10^− 4^
γ_GB_AIMD_ (mJ/m^2^)	1547	1126	812	1.643
Variance, *F* (eV)	0.103	2.831	0.070	2.345
γ_GB_ DFT 0 K (mJ/m^2^)	1436		656	

With the inclusion of dynamics, the GB atoms remain on average at the equilibrium positions obtained from static DFT and no reconstruction of the GBs were observed. While the test was done for the two Σ03 (111)[$\:\bar{1}$10] GBs because modelling the large GB supercells with AIMD is computationally unfeasible at present, the higher angle GB models are expected to be stable at finite *T* as previously demonstrated for other fcc materials^27^^,28^. The effect of phonons on the GB energies is more pronounced for UC as shown in the values of γ_GB_AIMD_. The difference in γ_GB_AIMD_ between 1700 and 298 K is of ≈ 6.8 eV for UN and of ≈ 9.5 eV for UC which indicates a larger phonon contribution to the γ_GB_AIMD_ of UC. This fact is also shown in the increase of γ_GB_AIMD_ for UC that occurs when going from 280 K to 1700 K, while the γ_GB_AIMD_ changes slightly in the opposite direction for UN. The stability of the GBs in the AIMD simulations shows that the CSL models here used can be employed to study properties of these GBs that do not depend on larger supercells such as large agglomerates of impurities, large vacancy clusters or cavities. Intensive properties such as point defects, volume expansion, GB energies and symmetry features can be correctly described with these GB models.

**Fig. 4:**
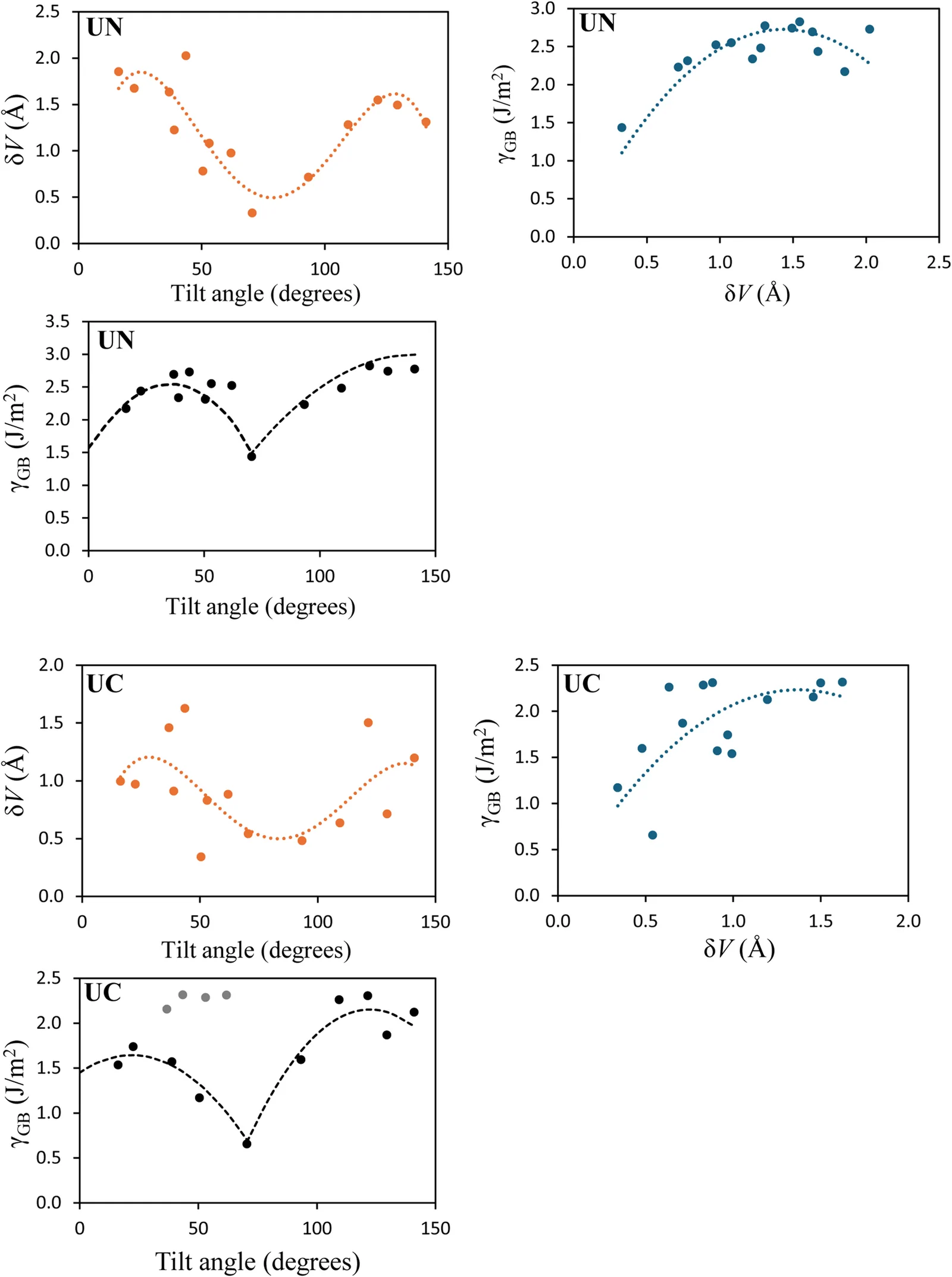
Correlations between tilt angle (degrees), excess volume (δ*V* (Å)) and GB energy (γ_GB_ (J/m^2^)) for the GBs here studied at 0 K for UN and UC. The corresponding fitting functions are given in Table 5. The statistical errors of the goodness-of-fit for each function are provided in the SI section.

The GB data calculated with static DFT and shown in Table 3 was used to study correlations between properties of the GBs such as symmetry features and other descriptors of their energy. Fitting functions were used for correlating the properties of the different data sets. The resulting data is shown in the collection of images of Fig. 4.

Both for UN and UC, the dependencies of γ_GB_with tilt angle tend to follow a trend similar to those found for other rock salt structure materials such as MgO^25^. In MgO, the Σ03 type GBs with tilt angles at around 70° are considerably more stable than the remaining. For the set of symmetric tilt GBs here studied, the 70° tilt angle is a global minimum in terms of resulting γ_GB_ and deviations from this angle in both directions lead to increased γ_GB_ and consequently lower stability of those GBs when compared to the Σ3. For both UN and UC, the GBs with tilt angles at around 70° are also those with the smaller δ*V*.

**Table 5:** Fitting functions for describing different correlations between properties of the GBs: tilt angle (degrees), excess volume (δ*V* (Å)), GB energy (γ_GB_ (J/m^2^)) and correlation coefficients (R^2^). The statistical errors of the goodness-of-fit for each function are provided in the SI section.

	Correlation	Function	R^2^
UN	x = tilt angle y = δ*V*	y = (−1.73 × 10^− 7^)x^4^ + (5.36 × 10^− 5^)x^3^ - (5.30 × 10^− 3^)x^2^ + (1.76 × 10^− 1^)x	0.714
	x = δ*V* y = γ_GB_	y = −1.32 × ^2^ + 3.79x	0.720
	x = tilt angle y = γ_GB_	for tilt angle < 70°: y = (−8.00 × 10^− 4^)x^2^ + (6.00 × 10^− 2^)x + 1.54	0.67
		for tilt angle > 70°: y = (−3.00 × 10^− 4^)x^2^ + (8.48 × 10^− 2^)x − 2.99	0.98
UC	x = tilt angle y = δ*V*	y = (−7.85 × 10^− 8^)x^4^ + (2.60 × 10^− 5^)x^3^ - (2.78 × 10^− 3^)x^2^ + (1.02 × 10^− 1^)x	0.343
	x = δ*V* y = γ_GB_	y = −1.19 × ^2^ + 3.26x	0.399
	x = tilt angle y = γ_GB_	for tilt angle < 70°: y = (−4.00 × 10^− 4^)x^2^+ (1.76 × 10^− 2^)x + 1.45	0.952
		for tilt angle > 70°: y = (−5.47 × 10^− 4^) x^2^ + (1.34 × 10^− 1^)x −6.04	0.914

In fcc materials, the atomic coordination in intrinsic stacking faults and also their excess volume, resemble often both the structure of symmetric tilt GBs and the excess volume of GBs for GBs with tilt angles ≤ 50.48°^73^. For UN the function that fits the correlation between γ_GB_ and tilt angle for tilt angle < 70° converges naturally to the stacking fault energy at 0° tilt angle according to the model suggested in Sect. 2.5 of this work. The obtained stacking fault energy value of 1.57 J/m^2^ for UN is in excellent agreement with the literature data, 1.54 J/m^2^^80^. For UC, the corresponding correlation was forced to ignore the 4 GBs that have the higher energies at tilt angles < 70° show in Fig. 4 and that are outside of the fitting function. These GBs are the: Σ17 (530)[001]; Σ29 (520)[001]; Σ05 (210)[001] and Σ05 (310) [001]. These defects possess some of the more open GB structures here studied and their high γ_GB_ for UC agrees with previously published^25^ observations and with the fact that also for UN these are the highest energy GBs at angles < 70°. Additionally, as shown in Fig. 4, these four GBs have tilt angles very similar to other GBs that have considerably lower γ_GB_ which implies that the GBs with lower γ_GB_ are expected to be those that will occur more frequently^63^^,81^. Forcing the fitting function to ignore these 4 high energy GBs for UC allowed the fitting function to converge to a reasonable stacking fault energy at 0° tilt angle which is equal to 1.45 J/m^2^. At present there is no literature data for the stacking fault energy of UC but its value is expected to be similar to that of UN. Thus, the value here obtained can be a good approximation.

The quantity δ*V* is often described as a first good indicator of relative γ_GB_ and relative stability of GBs in materials. In these analyses, the GBs with smaller δ*V* are suggested as the most stable due to lower γ_GB_. However, the GBs in many materials do not obey a trend where their energies can be easily predicted from their δ*V*, and even for simple materials, complex correlations between these two quantities have been described^21^. For UN and UC, the data of Fig. 4 shows that when correlated with the tilt angle, both γ_GB_ and δ*V* have minima at angles of 70° and around this value. This makes the correlation between γ_GB_ and δ*V* also shown in Fig. 4 be single-valued and of the simple parabolic type. This indicates that the δ*V* can be used as a predictor of the GB energy especially for UN where the correlation between these two properties is strong with R^2^ = 0.720. For UC the correlation coefficient of around 0.4 indicates that δ*V* can be used as an indication of the relative γ_GB_ with careful considerations because of the lower correlation coefficient. Factors particular to this material and that are not investigated here could be responsible for the worse correlation coefficient. Whenever the statistical significance allows, the prediction of the γ_GB_ from δ*V* can allow the prediction of the relative frequency of occurrence of the GBs.

## Conclusions

A set of 14 symmetric tilt GBs was studied for UN and UC with DFT using PBE after tests of its performance against three DFT functionals in the study of the bulk, including the magnetic properties of these materials. For bulk UN, we found that DFT + U predicted the correct magnetic configuration, the AFM, as lowest in energy, but the deviation between the predicted magnetic moments in U from the experimental data are large, in the order of 50%. The PBE functional predicted the FM configuration as lower in energy when compared to the AFM, but with a very small energy difference. The difference in magnetic moment of U in UN between the PBE and the experimental data is 2.8%. The inclusion of the f-orbitals in the one-center PAW charge densities that are used in the charge density mixer, using non-collinear unconstrained spin optimization routines, simultaneously with optimization of the lattice parameters, are important aspects for the determination of reliable magnetic properties.

Our data shows that from the GB set studied, Σ03 GBs are the lowest in energy for both UN and UC. The correlations between properties of the GBs such as tilt angle, excess volume and energies show that the Σ03 are the most stable with a global minimum around the tilt angle of 70°. The plot of the tilt angles vs. GB energies converges naturally to the previously published stacking fault energy of UN at the tilt angle of 0° which shows that the set of GBs used here is a good approximation to describe the GBs that occur in the real material. For UC the corresponding correlation was forced to ignore four GBs with large cavities that have considerably large energies, a fact that agrees with previous observations for other materials. Using the correlation between tilt angles vs. GB energies we obtained a stacking fault energy for UC. The correlation between GB excess volume and GB energy is strong for UN and shows that the excess volume can be a good predictor of GB energy. For UC the corresponding correlation is weaker and predictions of GB energy from GB excess volume should be done with careful consideration. Similarly to what was previously observed for MgO which also has rock salt structure, this set of GBs is expected to be a good description of CSL GBs that occur in microstructures close to or in equilibrium.

## Supplementary Information

Below is the link to the electronic supplementary material.


Supplementary Material 1


## Data Availability

The data generated is available from the corresponding author upon reasonable request.
