# Supplementary material for: Symmetric tilt grain boundaries in uranium nitride and uranium carbide
Source: Sci Rep. 2026 Aug 31;16:27231. doi: 10.1038/s41598-026-67229-y (PMC13529789; doi:10.1038/s41598-026-67229-y)
Supplement: Supplementary file 1 [file 41598_2026_67229_MOESM1_ESM.docx]

**Supplementary Information for**

**Symmetric Tilt Grain Boundaries in Uranium Nitride and Uranium Carbide**

Cláudio M Lousada^1^

1) Department of Materials Science and Engineering, KTH Royal Institute of Technology, SE-100 44 Stockholm, Sweden

*	Correspondence: cmlp@kth.se;

1. **Error analyses and statistics for evaluation of the Goodness-of-Fit for the functions that correlate different GB properties**

All functions shown in Figure 4 and described in Table 5 have been fit to the corresponding discrete data with the MATLAB R2024b software.^1^ The polynomial curve fitting module was used for searching for the best fitting functions and for the statistical analysis of the Goodness-of-Fit. The functions fit are polynomials of the type

$f\left( x \right)={p_{n}x}^{n}+{p_{n-1}x}^{n-1}+\ldots+p_{0}$	(SI1)

where the number of indeterminates depends on the data to which the fit function is applied to. The goodness-of-fit statistics obtained from MATLAB for each of the functions shown in Figure 4 and given in Table 5 are given in Tables SI1 and SI2 below.

The acronyms and symbols here shown follow the nomenclature of MATLAB and are typical of correlation statistics^2^ and defined as follows: SSE - sum of squares due to error (which is the least-squares error of the fit); R^2^ - square of the correlation between the response values and the predicted response values; Adj R^2^ - degrees of freedom adjusted R-square; RMSE - root mean squared error or standard error.

Table SI1. Goodness-of-fit statistics for the correlations between GB data shown in Figure 4 and Table 5 devised for the set of UN.

| Goodness-of-fit statistics | | | | |
| --- | --- | --- | --- | --- |
| Correlation | ***x* = tilt angle**  ***y* = δ*V*** | ***x* = δ*V***  ***y* = γ_GB_** | ***x* = tilt angle < 70°**  ***y* = γ_GB_** | ***x* = tilt angle > 70°**  ***y* = γ_GB_** |
| SSE | 0.8266 | 0.7122 | 0.7355 | 0.0214 |
| R^2^ | 0.714 | 0.720 | 0.670 | 0.980 |
| Adj R^2^ | 0.628 | 0.5374 | 0.3112 | 0.9742 |
| RMSE | 0.2875 | 0.2436 | 0.3501 | 0.0845 |

Table SI2. Goodness-of-fit statistics for the correlations between GB data shown in Figure 4 and Table 5 devised for the set of UC.

| Goodness-of-fit statistics | | | | |
| --- | --- | --- | --- | --- |
| Correlation | ***x* = tilt angle**  ***y* = δ*V*** | ***x* = δ*V***  ***y* = γ_GB_** | **x = tilt angle < 70°**  **y = γ_GB_** | **x = tilt angle > 70°**  **y = γ_GB_** |
| SSE | 1.3227 | 2.0469 | 0.0358 | 0.1561 |
| R^2^ | 0.343 | 0.399 | 0.952 | 0.914 |
| Adj R^2^ | 0.1438 | 0.3275 | 0.9037 | 0.856 |
| RMSE | 0.3637 | 0.4130 | 0.1338 | 0.2281 |

**References**

1. T. M. Inc., The MathWorks Inc.2022.

2. A. G. Asuero, A. Sayago and A. G. González, *Critical Reviews in Analytical Chemistry*, 2006, **36**, 41-59.
